# Use of Ancillary Procedures in Combination with the MACS-Lift for Facial Rejuvenation: A Retrospective Cohort Study

**DOI:** 10.1007/s00266-025-05143-w

**Published:** 2025-09-04

**Authors:** Pehr Sommar, Jenaleen Law, Janne Ongena, Alexis Verpaele, Patrick Tonnard

**Affiliations:** 1 Nordiska Kliniken, Stockholm, Sweden; 2 Department of Molecular Medicine and Surgery, Karolinska Institutet, Stockholm, Sweden; 3 Department of Plastic, Reconstructive and Maxillofacial Surgery, St Vincent’s Hospital, Sydney, Australia; 4 Royal Australasian College of Surgeons, Sydney, Australia; 5 Department of Dermatology, AZ Sint Lucas, Ghent, Belgium; 6 Coupure Centre for Plastic Surgery, Coupure Rechts 164 c-d, 9000 Ghent, Belgium

**Keywords:** Facelift, MACS-lift, Necklift, ancillary procedures, Facial rejuvination

## Abstract

**Background:**

While facelifts remain central to facial rejuvenation, ancillary procedures are essential for addressing aspects of aging not corrected by facelifting alone, such as soft tissue atrophy and skin quality. Despite their routine use, few reviews describe their role alongside facelifts in modern practice.

**Objective:**

To define the range of ancillary procedures used with the Minimal Access Cranial Suspension (MACS) lift in current practice.

**Methods:**

A retrospective review was conducted on all MACS-lift procedures performed by the senior authors from January 1, 2018, to December 31, 2024. Data collected included demographics, surgical and ancillary procedures, and complications.

**Results:**

The MACS-lift technique has evolved to include deep neck reduction, centrofacial lipofilling, and skin resurfacing. Among 380 patients (356 females, 24 males), 81.8% underwent a primary facelift. Ancillary procedures were performed in 98.9% of cases, with an average of 5.8 procedures per patient, with this average being higher for female than for male patients (6.0 vs 4.5), and higher for patients 55 years or older than for younger patients (6.17 vs 5.13). The most common ancillary procedures were lipofilling (96.3%), brow lift (72.6%) neck lift (67.9%), nanofat microneedling (64.7%), blepharoplasty (57.4%), and lip lift (27.6%). Mean operative time was 3.93 hours. No major complications occurred. Minor complications included neuropraxia (4.7%), infection (2.9%), skin necrosis (2.9%), sialoma (2.6%), seroma (2.4%), hematoma (1,6%) and wound healing disturbances (1,3%).

**Conclusions:**

Ancillary procedures are frequently and safely combined with the MACS-lift to address facial ptosis, volume loss, and skin aging, enhancing overall rejuvenation outcomes.

**Level of Evidence IV:**

This journal requires that authors assign a level of evidence to each article. For a full description of these Evidence-Based Medicine ratings, please refer to the Table of Contents or the online Instructions to Authors www.springer.com/00266.

## Introduction

Optimal facial rejuvenation outcomes require a comprehensive approach that addresses the three principal components of facial aging: 1. sagging of facial tissues, 2. deflation of facial volume, and 3. skin aging [1]. While a facelift remains the gold standard of facial rejuvenation, ancillary procedures are integral to address aging processes that are not addressed by a facelift alone. These ancillary procedures include lipofilling to treat volume loss, skin resurfacing to improve skin quality, and other procedures to address brow, eyelid, or lip ptosis. Despite the widespread use of ancillary procedures, there is a paucity of reviews detailing their combined use with facelifts in current practice.

### Treating Facial Ptosis

**Fig. 1:**
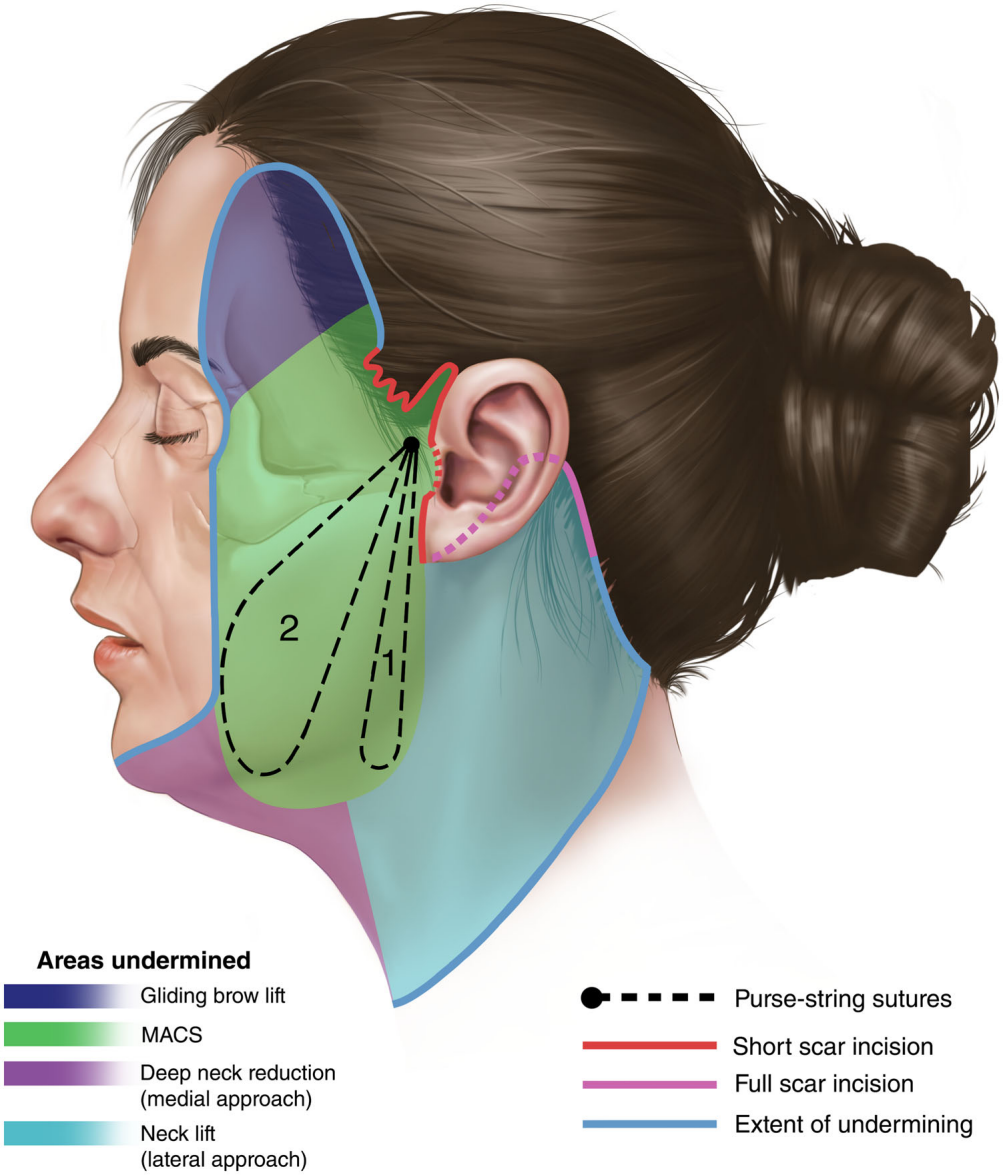
MACS-lift technique is utilizing suspension sutures to lift and reposition the SMAS and deeper facial tissues. Schematic drawing of incision, extent of undermining and placement of purse-string sutures in a MACS-lift and necklift.

Facelift remains the cornerstone of treating ptosis of facial tissues [2]. The Minimal Access Cranial Suspension (MACS) lift was introduced in 2002, and the senior authors have treated over 2500 patients with this facelift technique [3]. The facial skin is undermined in the subcutaneous plane, and two purse-string sutures through the superficial musculoaponeurotic system (SMAS) are used to reposition and elevate the facial tissues to achieve a lifting effect (Figure 1) [3–5]. Since its introduction, the MACS-lift technique has continued to evolve. The skin undermining is now more extensive, often extending to the lateral canthus and beyond the zygomatic and mandibular retaining ligaments, to enhance the effects of the purse-string sutures.

In the original MACS-lift description, the aging neck was treated with a suture to suspend the platysma in a vertical vector, in combination with liposuction to treat the central neck [3, 6]. Since 2018, the senior authors have adopted the deep neck lift which includes resection of subplatysmal fat, shaving of the anterior belly of digastric muscle, resection of the superficial lobe of the submandibular glands, midline platysmarraphy, and horizontal transection of platysma.

Several areas such as the forehead, eyelids, and perioral region are hardly improved during facelift surgery. Additional surgical procedures such as brow lift, blepharoplasty, and lip lift help address these areas. The Fogli temporal lift was the preferred brow lift for the senior authors, but this has been replaced by the Gliding Brow Lift (GBL) since 2019 [7, 8]. The GBL and a hemostatic net is often performed concomitant and blends well with the MACS-lift to correct lateral brow hooding as both dissections are in the subcutaneous plane [9].

### Treating Facial Volume Deflation

Lipofilling was routinely incorporated with the MACS-lift procedure since 2004. Microfat, harvested with a 2.4 mm diameter cannula with 20 sharpened 1mm holes, is used to treat facial volume loss in the temporal, infrabrow, tear trough, malar areas, and the nasolabial and marionette grooves. Unlike synthetic fillers, autologous fat is not associated with hypersensitivity reactions or granuloma formation and has potential regenerative properties such as promoting collagen formation [10].

### Treating Skin Aging

Skin rejuvenation can be achieved using several methods, including laser, chemical peels, injectables, and topical treatments [11]. Facial resurfacing procedures employed by the senior authors included CO2-laser with a later transition to Erbium YAG-laser which produced less hypopigmentation and erythema. For deeper rhytids, croton oil peel is used except in undermined areas of the MACS-lift [12]. Over the recent years, the senior authors have preferred croton oil peel for skin resurfacing due to the superior clinical results seen.

Since 2008, the senior authors began treating deep rhytids using Sharp Needle Intradermal Fat grafting (SNIF), often in conjunction with other skin resurfacing techniques [13]. This involves dermal injection of microfat into rhytids usually in the forehead, perioral and neck areas. SNIF fills the rhytids internally, and has a synergistic effect with skin resurfacing procedures such as a chemical peel and lasers which treat rhytids externally [1]. Another strategy to improve skin quality and texture is to use nanofat, the stromal vascular fraction of fat that is processed through mechanical emulsification of microfat [14, 15]. Nanofat is routinely used by the senior authors in conjunction with Dermapen® microneedling at the end of the MACS-lift to add stem cells and regenerative elements to the skin and hence improve skin quality and texture [16–18]. As the clinical effects of nanofat takes 6-9 months to be visible, we add hyaluronic acid (1cc), vitamin C (1cc), and botulinum toxin (50 units) to the nanofat (8cc) to give an instant boosting effect on the skin. After the microneedling, residual nanofat is mixed with cetomacrogol and applied three times daily to the skin until it has healed. Patients are also encouraged to use topical tretinoin (retinoic acid) and l-ascorbic acid (Vitamin C) to improve skin quality [11], commencing several weeks before surgery.

## Methods

The purpose of this study was to define the spectrum of ancillary procedures we currently use in combination with the MACS-lift procedure. We performed a retrospective review of all patients who underwent a MACS-lift procedure by the senior authors over the past seven years, between 1 January 2018 and 31 December 2024. Demographic information including age and gender were retrieved from patient records. Clinical information was collected on facelift (primary or secondary), neck rejuvenation (liposuction, lateral skin-platysma displacement [LSD], deep neck lift), ancillary surgical procedures (brow lift, blepharoplasty, lip lift), and non-surgical procedures (lipofilling, SNIF, laser, chemical peeling, topical retinoic acid and l-ascorbic acid). Any complications were recorded.

## Results

### Demographics

A total of 380 patients underwent a MACS-lift by the senior authors between 1 January 2018 and 31 December 2024. Of the 380 patients, 356 (93.7%) were women, 24 (6.3%) were men, and the average age was 59 years old (range 37 to 88). Most patients underwent a primary MACS-lift (*n*=311, 81.8%) rather than a secondary MACS-lift (*n*=69, 18.2%) (Table 1).

**Table 1:** Patient demographics

Characteristic	Average	Range	Number (%)
Age (years)	59	37–88	
Total individuals			380 (100)
Females			356 (93.7)
Males			24 (6.3)
Primary facelift			311 (81.8)
Secondary facelift			69 (18.2)

### Ancillary Procedures

Almost all patients had ancillary procedures performed with their MACS-lift (*n*=376, 98.9%). The average number of ancillary procedures for each patient was 5.8 (range 0 to 10). The average number of ancillary procedures undertaken by female patients was higher than for male patients (6.0 vs 4.5). Patients who were 55 years or older (*n*=271) had more ancillary procedures than patients younger than 55 years old (*n*=109) (average of 6.17 vs 5.13). The total duration of surgery for the MACS-lift and ancillary procedures involving the face was 3.93 hours on average (range 1.75 to 7 hours). See Table 2 for the types of ancillary procedures performed.

**Table 2:** Procedure details

**Total individuals**	**380**	
**Characteristic**	**Number (%)**	**Mean cc (Range)**
MACS-lift with no ancillary procedures	4 (1.1)	
MACS-lift with ancillary procedures	376 (98.9)	
**Autologous fat injection to face**	366 (96.3)	
Micro fat graft for volume	353 (92.9)	19.4 (2–61.4)
SNIF for rhytids	324 (85.3)	4.3 (0.3–22)
**Brow lift**	276 (72.6)	
Fogli temporal lift	64 (16.8)	
Gliding brow lift	212 (55.8)	
**Blepharoplasty**	218 (57.4)	
Upper blepharoplasty	141 (37.1)	
Lower pinch blepharoplasty	132 (34.7)	
Transconjunctival lower lid fat resection	57 (15.0)	
**Lip lift**	105 (27.6)	
**Cervicoplasty**	258 (67.9)	
Subplatysmal fat resection	107 (28.2)	
Submandibular gland resection	95 (25.0)	
Digastric muscle resection	52 (13.7)	
Platysma transection	201 (52.9)	
Midline platysmarraphy	81 (21.3)	
Digastric plication	33 (8.7)	
**Skin rejuvenation**	283 (83.5)	
Laser	90 (23.7)	
Peeling	46 (12.1)	
Topical retinoic acid	233 (61.3)	
Topical l-ascorbic acid	225 (59.2)	
Nanofat microneedling	246 (64.7)	
**Hemostatic net**	273 (71.8)	
**Additional procedures**	85 (22.4)	

### Lipofilling

Most patients (*n*=353, 92.9%) underwent lipofilling with microfat at the time of their MACS-lift to treat facial volume loss. The mean injected volume of microfat was 19.4 cc (range 2 to 61.4 cc). For male patients, the mean injected volume of microfat was higher (23.4 cc) than for female patients (19.0 cc). Most patients (*n*=324, 85.3%) had SNIF to efface deep rhytids. The mean volume of microfat used for SNIF was 4.3 cc (range 0.3 to 22 cc). SNIF was mostly performed for perioral, glabellar, and neck rhytids.

### Brow Lift, Periorbital and Lip Rejuvenation

Of the 380 patients undergoing MACS-lift, 276 (72.6%) patients had a concomitant brow lift. The brow lift technique employed was a Fogli technique (*n*=64, 16.8%) or a Gliding Brow Lift (GBL) (*n*=212, 55.8%). The GBL was adopted from November 2019 when this technique was introduced [8]. Blepharoplasty was performed in 218 (n=57.4%) patients, and involved upper blepharoplasty (*n*=141), lower pinch blepharoplasty (n=132) and transconjunctival lower lid fat resection (*n*=57). Lip lift was performed in 105 (27.6%) patients to correct age-related lip lengthening.

### Neck Rejuvenation

Of the 380 patients undergoing MACS-lift, 258 (67.9%) patients had additional neck work other than submental or jowl liposuction. Deep neck work was performed in 145 (38.2%) patients. Of the 380 patients, 107 (28.2%) patients had subplatysmal fat resection, 95 (25.0%) had submandibular gland resection, 52 (13.7%) had anterior belly of digastric muscle shave. Platysma transection was performed in 201 (52.9%) patients, and midline platysmarraphy was performed in 81 (21.3%) patients, and digastric plication was performed in 33 (8.7%) patients.

The seroma/sialoma rate in the neck was 5% (*n*=19) and of these patients, 13 (68.4%) patients underwent deep neck work. However, 8 of these 13 patients who had a seroma/sialoma after deep neck work did not have a neck drain. All cases were successfully treated with aspiration.

### Skin Resurfacing Procedures

Of the 380 patients undergoing MACS-lift, 90 (23.7%) patients had laser resurfacing and 46 (12.1%) patients had a croton oil peel. No patient who received a croton oil peel suffered any adverse cardiotoxicity. Two-thirds of patients were using topical retinoic acid (n=233, 61.3%) and l-ascorbic acid (n=225, 59.2%) serums or creams before and after surgery. Two-thirds of patients (*n*=246, 64.7%) underwent microneedling with nanofat at the end of the MACS-lift procedure.

### Other Surgical Procedures

Of the 380 patients, 85 (22.4%) patients received additional procedures during the MACS-lift surgery not directly related to facial rejuvenation. The procedures were breast surgery (n=25), rhinoplasty (*n*=15), lipofilling of hands (*n*=12), brachioplasty (*n*=11), abdominoplasty (*n*=8), buccal fat pad removal (*n*=4), nanofat microneedling of the decolletage (*n*=3), scar revision (*n*=2), earlobe correction (*n*=8), otoplasty (*n*=3), and eyelid ptosis correction (*n*=1).

### Complications Within 30 Days

No major complications were reported. Minor complications include temporary neuropraxia (4.7%), minor infection (2.9%), skin necrosis (2.9%), sialoma (2.6%), seroma (2.4%), hematoma (1.6%), and other wound healing disturbances (1.3%) (Table 3). All cases of skin necrosis eventually healed by secondary intention with acceptable scarring. Seromas were treated with aspiration. Sialomas were successfully treated with supportive measures including aspiration, botox injection, antibiotics, compresses, and dietary modifications.

**Table 3:** Complications within 30 days

Total individuals	380
Characteristic	Number (%)
Neuropraxia	18 (4.7)
Minor infection	11 (2.9)
Skin necrosis	11 (2.9)
Sialoma	10 (2.6)
Seroma	9 (2.4)
Haematoma	6 (1.6)
Wound healing disturbances	5 (1.3)

## Discussion

### Ancillary Procedures

Facial aging consists of sagging of facial tissues, volume deflation, and skin degeneration. A modern approach to facial rejuvenation should address all these changes with ancillary procedures to amplify the facelift results. Stein and Aston (2023) currently incorporate dermabrasion, lasers, chemical peels, and radiofrequency devices during their facelift operations [2]. Like our practice, Rohrich and Mohan (2019) use lipofilling to treat volume deflation and use chemical peels and lasers for skin resurfacing. They also use multiple modalities to target age-related changes in specific anatomic regions, such as the chin, earlobe, hand, perioral area, and the lower eyelid-cheek junction [19]. Anlatici et al (2018) performed a retrospective review of 203 patients and reported combining their facelifts with genioplasty, fat injections, upper and lower blepharoplasty [20]. There is otherwise limited data describing the spectrum, frequency, and combination of ancillary procedures currently used in modern facelift practices.

Some authors performed ancillary procedures in a separate session to limit the surgical time [21]. However, we performed ancillary procedures in the same operating session as the MACS-lift with a total operating time of 3.93 hours on average. Evidence suggests that both SMAS and deep plane facelifts produce good aesthetic results [22], but the MACS-lift has the advantage of a short surgical time even with the addition of ancillary procedures.

### Treating Facial Ptosis

The MACS-lift has evolved since its original description [3] and now involves more extensive undermining, often reaching the lateral canthus, and extends beyond the zygomatic and mandibular retaining ligaments. By medializing the SMAS imbrication closer to the mobile facial tissues, a more effective and long-lasting plication can be achieved without a deep plane approach [23].

In addition to a MACS-lift, we currently perform a deep neck reduction if the patient has fullness of the neck. Two-thirds (67.9%) of our MACS-lift patients also underwent a neck lift. This is compared with our earlier cohort of 450 MACS-lift patients (1999-2005) where only 5.1% of patients underwent additional neck procedures [6]. The shift in our practice toward deep neck work can be partially explained by our later cohort of patients being older in age, and the higher number of secondary facelifts performed, where the neck is often a target for rejuvenation. The neck has also received increasing attention over the years and an increasing number of patients are seeking neck rejuvenation to match the results obtained in the face [24].

The introduction of deep neck work to our practice began in 2018 using the Pelle Ceravolo LSD technique [25] and occasionally the Jacono and Botti techniques [26, 27]. Although a lateral approach enables submandibular gland resection to be performed, a submental incision is required to reduce central volume in obtuse necks. Resection of the submandibular gland can be controversial due to increased risk of bleeding and sialoma [28] but we believe that gland reduction is necessary to achieve a satisfactory aesthetic result for patients with obtuse necks. A quarter of our patients underwent submandibular gland resection, which is comparable to other studies [23, 28]. Our sialoma rate was 2.6%, compared to the rates of 0 to 24% in other studies [29, 30]. Nearly all our patients who experienced sialoma were from our earlier cohort (9 out of the 10 patients were from 2022 or earlier). We believe our recent practice of using LigaSure® and Shalya® to seal the submandibular gland during resection has significantly improved our sialoma rates. In a recent review of 83 patients undergoing deep plane neck lifts, Basaran and Comert (2025) used LigaSure for submandibular gland excisions. They had no cases of sialoma, and concluded that LigaSure-assisted submandibular gland excision was easier, faster, and also associated with lower rates of bleeding and hematoma [29]. In addition, 8 of the 10 patients who had a sialoma did not have a neck drain, thus highlighting the importance of using neck drains with any component of a deep neck reduction. In addition to submandibular gland resection, reducing the digastric muscles and subplatysmal fat helps achieve a smooth neck contour [24].

**Fig. 2:**
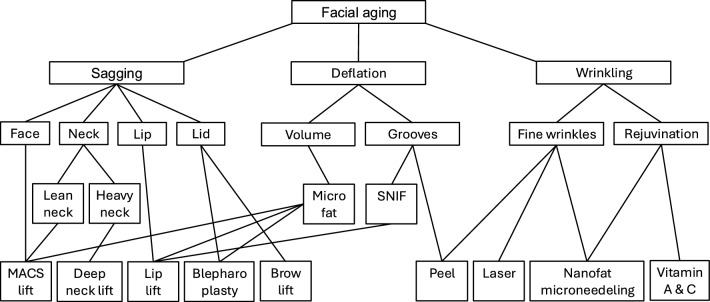
Flowchart illustrating ancillary procedures for facial rejuvenation.

A lip lift is performed in the presence of age-related upper lip ptosis when the patient no longer has upper teeth show when the lips are slightly parted. There should typically be 2–3 mm of upper incisor show in repose [19]. For perioral rejuvenation, we used various techniques including lip lift, croton oil peeling, laser, microfat, SNIF to treat lip ptosis and perioral rhytids. See Figure 2 for our treatment algorithm.

**Fig. 3:**
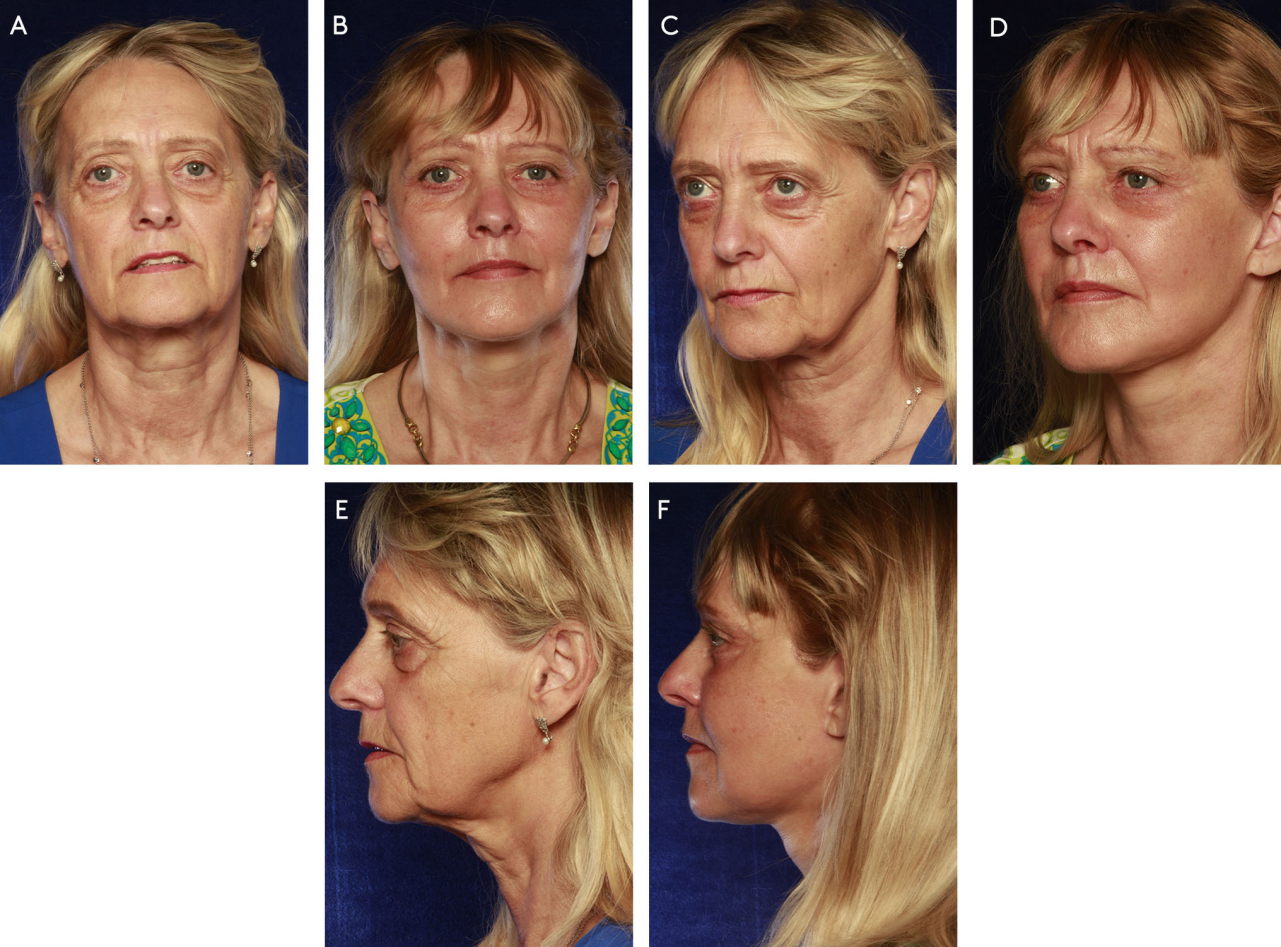
A 63 year-old female presented with severe laxity and wrinkling of facial skin and neck, drooping of the tail of the eyebrow, upper eyelid laxity and deflation of the malar, periorbital and perioral area. She was treated with a MACS-lift, LSD neck lift with submandibular gland resection, liplift, upper blepharoplasty, transconjunctival lower lid fat resection and lower pinch blepharoplasty, microfatgrafting (upper eyelid, malar, nasolabial folds, marionette lines), SNIF (white roll, philtrum, perioral, glabella), full face and neck nanofat microneedeling, hemostatic net. Preoperative photos (**A**, **C**, **E**) and photos 4 years after surgery (**B**, **D**, **F**).

**Fig. 4:**
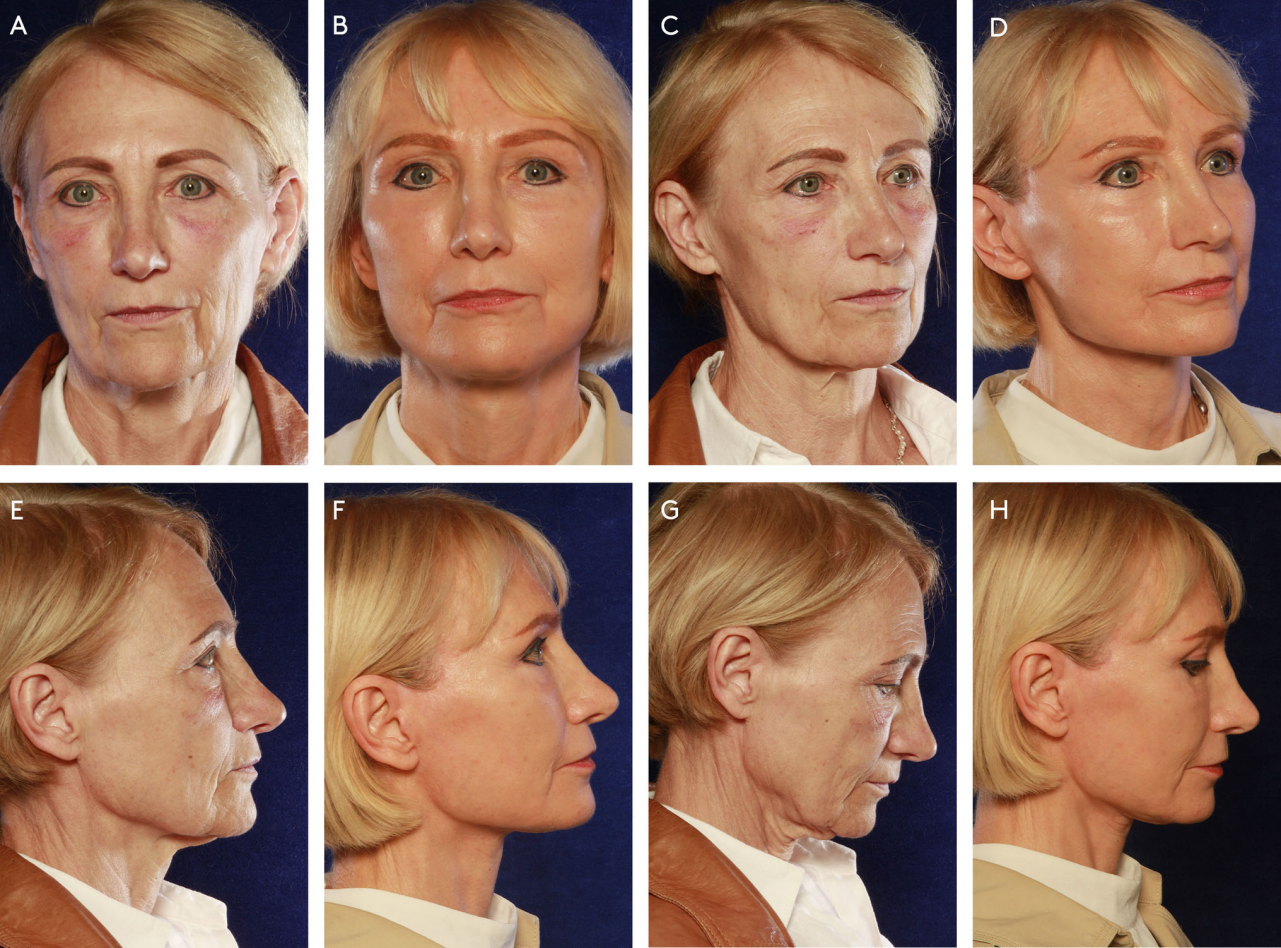
A 66 year-old female presented with jowling and laxity of the neck tissues, perioral rhytids and marionette grooves, deflation of the midface and upper eyelid laxity. She was treated with a MACS-lift, neck lift with midline platysmarraphy and platysma transection, upper blepharoplasty, lower pinch blepharoplasty, gliding brow lift, microfatgrafting (upper eyelid, malar, nasolabial folds, marionette lines, lips), SNIF (white roll, perioral, glabella, neck wrinkles), full face and neck nanofat microneedeling, hemostatic net. Preoperative photos (**A**, **C**, **E**, **G**) and photos 1 year after surgery (**B**, **D**, **F**, **H**).

**Fig. 5:**
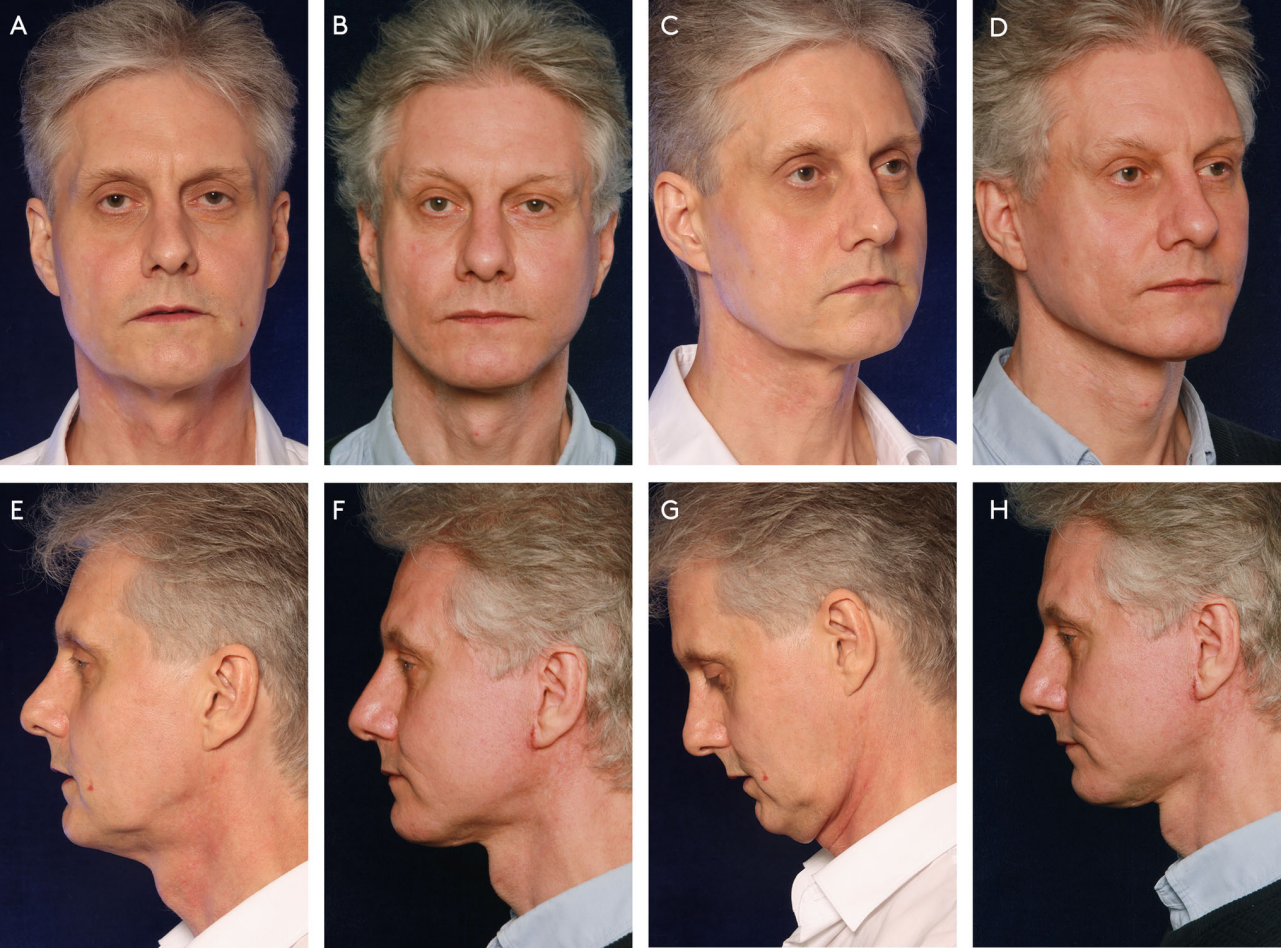
A 54 year-old male presented with jowling, laxity of the neck tissues with accumulation of volume, drooping of the tail of the eyebrow, deflation of the periorbital and perioral area, rhytids in the perioral area and glabella. He was treated with a MACS-lift, liposuction neck, central deep neck work (subplatysmal fat excision, submandibular gland resection, midline platysmarraphy, platysma transection), gliding brow lift, microfatgrafting (upper eyelid, malar), SNIF (perioral, philtrum, glabella), full face and neck nanofat microneedeling, hemostatic net. Preoperative photos (**A**, **C**, **E**, **G**) and photos 1 year after surgery (**B**, **D**, **F**, **H**)

The GBL is offered to patients with signs of lateral brow hooding, and blends well with the MACS-lift as both dissections are in the subcutaneous plane [8]. Upper blepharoplasty is offered to patients with dermatochalasis. Pinch lower blepharoplasty is performed if there is excess lower eyelid skin which is further aggravated with a vertical vector facelift. Transconjunctival fat resection is offered to patients with significant fat herniation. For harmonious periorbital rejuvenation, most patients undergoing blepharoplasty received lipofilling to the infrabrow and malar areas to minimize periorbital hollowing and to blend the lid-cheek junction (Figs. 3, 4 and 5).

### Treating Facial Volume Deflation

According to Marten and Elyassnia (2018), lipofilling is one of the most powerful additions to facelift surgery and produces a long-lasting rejuvenation that cannot be attained by either modality alone [31]. Their usual lipofilling treatment sites were similar to ours and included the temporal, malar, periorbital areas, as well as the chin, lips, pre-jowl sulcus, and the nasolabial and marionette grooves [31]. In a meta-analysis of 1568 patients who underwent facial rejuvenation with lipofilling, there were high levels of patient (91.1%) and surgeon (88.6%) satisfaction even despite some graft resorption [32]. Lipofilling with microfat was used in nearly all of our patients to address volume deflation and should be considered an integral part of modern facelift surgery. Microfat is created by liposuctioning with a multiport cannula with small 1mm holes. Microfat distributes more evenly and is less prone to creating lumps in delicate areas of the face including the eyelids and lips [33]. Microfat has been shown to contain viable adipocytes and adipose-derived stem cells (ASCs) [10]. ASCs can promote new collagen formation and dermal hyperplasia, and may thus offer additional tissue regeneration [10].

### Treating Skin Degeneration

Although skin treatments have traditionally been neglected by plastic surgeons, we find that virtually all patients in our practice can improve on their skin quality. In our current practice, patients are routinely offered topical tretinoin and l-ascorbic acid serum or cream, and nanofat microneedling. The patients with deeper rhytids are offered croton oil peeling. Unlike older formulations of the croton oil peel, newer versions are suitable for all ages and skin types with less concern of hypopigmentation [34]. There are concerns of cardiotoxicity with using croton oil peels, but this did not affect any of our patients. It is advised to have cardiac monitoring of the patient during the procedure and to wait 10–15 minutes between treating each cosmetic unit to minimize cardiotoxicity [34].

### Complications

All cases of skin necrosis in our patients healed conservatively with acceptable scarring, and this only affected patients undergoing combined face and neck lifts with extensive skin undermining. Tranexamic acid was used in the MACS-lift infiltration solution between 2018 and 2023 but is now omitted after reports of higher rates of skin necrosis with its use [35].

### Study Limitations

The limitations of this study is the retrospective design and thus the lack of patient reported outcome measures, which may be of interest to future studies.

## Conclusions

The MACS-lift is our cornerstone of treating facial aging and the technique continues to evolve. In addition to the MACS-lift, ancillary procedures are integral to address all aspects of facial aging. The results of a facelift can be synergistically improved by volumizing regions of the face that have undergone fat atrophy, and skin resurfacing techniques can improve skin quality (pigmentation, wrinkling, and laxity). The MACS-lift and ancillary procedures can be used in combination effectively, and without major complications, to meet the diverse needs of individuals seeking facial rejuvenation. Our results show that the MACS-lift and ancillary procedures can be undertaken in a short time frame, which is a benefit of the MACS-lift in comparison with more extensive deep plane procedures.
